# Deletion of the predicted β-(1,3)-glucan synthase gene *bgs1* changes the cell wall composition and impacts growth and fruiting body formation of *Schizophyllum commune*

**DOI:** 10.1016/j.tcsw.2026.100183

**Published:** 2026-10-16

**Authors:** Ella M. Schunselaar, Fleur E.L. Kleijburg, Adil A. Safeer, Ajit K. Bishoyi, Sophie J.L. van Velzen, Peter Jan Vonk, Luis G. Lugones, Marc Baldus, Han A.B. Wösten

**Affiliations:** a Microbiology, Department of Biology, Utrecht University, Padualaan 8, Utrecht, 3584 CH, the Netherlands; b NMR Spectroscopy, Bijvoet Center for Biomolecular Research, Utrecht University, Padualaan 8, Utrecht, 3584 CH, the Netherlands

**Keywords:** Fungus, *Schizophyllum commune*, β-(1,3)-glucan, β-(1,3)-glucan synthase, Cell wall, Fruiting body formation

## Abstract

β-(1,3)-glucans are important for the integrity of the fungal cell wall and the interaction of fungi with the (a)biotic environment. For instance, the β-(1,3)(1,6)-glucan schizophyllan protects against thermal stress, starvation, and competition. Agaricomycetes like Schizophyllum commune have two β-(1,3)-glucan synthase (bgs) genes. Here, deletion constructs for the bgs1 and bgs2 genes were introduced in *S. commune*. A Δbgs2 strain was not obtained. Inactivation of bgs1 resulted in an approximately twofold lower production of water-soluble polysaccharide with FTIR characteristics consistent with schizophyllan. In addition, solid state NMR showed a relative 5-fold decrease in β-(1,3)-glucan / β-(1,3)(1,6)-glucan (schizophyllan) levels in the rigid part of the cell wall of the deletion strain, which was accompanied by a relative 3.3-fold and 2.7-fold increase in chitin and α-(1–3)-glucan, respectively. These changes in cell wall composition were accompanied by reduced growth, impaired fruiting body formation, and absence of spore production. Together, these data show for the first time the impact of a deletion of a β-(1,3)-glucan synthase gene in a mushroom-forming fungus on cell wall composition, growth, and fruiting body development.

## Highlights

• Inactivation of bgs1 reduces water-soluble polysaccharide production.
• Inactivation of bgs1 reduces relative β-(1,3)-glucan levels in the cell wall.
• bgs1deletion increases relative chitin and α-(1–3)-glucan levels in the cell wall.
• Inactivation of bgs1 impacts vegetative growth.
• Inactivation of bgs1 impacts fruiting body formation and abolishes spore production.

## 1 Introduction

*Schizophyllum commune* is a ubiquitous fungus that has been found on all continents (Kleijburg and Wösten, 2025). It is used as a model system to study mushroom development, mycelium materials, cell wall architecture, and the fungal immune system (Kleijburg and Wösten, 2025; Beijen et al., 2024, Beijen et al., 2025; van Maanen et al., 2026). The fact that *S. commune* can complete its life cycle in about ten days on defined media, that a high-quality genome sequence (Ohm et al., 2010a; Marian et al., 2022) and genetic tools (Ohm et al., 2010b; Vonk et al., 2019; Vonk and Ohm, 2021a) are available, and that its sexual reproduction provides a classical genetic tool to introduce or remove genetic traits facilitates the use of this fungus as a model system.

The life cycle of *S. commune* begins with the germination of a basidiospore into a homokaryotic mycelium (also called a monokaryon because each hyphal compartment contains a single nucleus). This homokaryon can form a fertile heterokaryon (also called a dikaryon because each hyphal compartment contains a nucleus of both parents) by fusing with a monokaryon with different *matA* and *matB* loci (Kües, 2015). The dikaryon grows vegetatively in the dark and at high CO_2_, while fruiting body formation is initiated by exposure to blue light and ambient CO_2_. Fruiting body formation is characterized by five stages (Kües and Navarro-Gonzalez, 2015). During stage I, hyphal aggregates of loosely intertwined hyphae are formed. These small hyphal knots develop into stage II aggregates, or stalks, by synchronous upwards growth of aerial hyphae, resulting in a tighter aggregate. Stage III primordia are formed when growth of the inner hyphae of the stalk is suppressed. As a result, the outer hyphae become longer than the inner hyphae. These longer outer hyphae grow inwards and then downwards into the center of the primordium, creating a curled perimeter surrounding a cavity in the middle. In stage IV, the primordia develop into young cup-shaped fruiting bodies. In doing so, the cavity of the stage III primordium widens, while the hymenium is formed at its bottom. The hymenium consists of a layer of palisade-shaped cells, from which basidia are formed that produce basidiospores. The young cup-shaped fruiting body can already sporulate but continues to grow in size. As the cup increases in size, the typical ‘split gills’ are formed. These are forked pseudolamellae that are formed by infoldings in the cup. The young fruiting bodies develop into stage V mature fruiting bodies by extending unilaterally. As a result, the outer hairy side of the pileus becomes the upper side and the inner hymenium faces down. The fruiting body obtains its typical fan-shape as it grows and additional secondary and tertiary pseudolamellae are formed. Fusion of the two nuclei occurs in the basidia of the mushroom, which is followed by the formation of a diploid zygote that undergoes meiosis, resulting in four haploid basidiospores. These basidiospores are dispersed (e.g. by air) and can start a new life cycle (Kleijburg and Wösten, 2025). *S. commune* can also form thick-walled chlamydospores but formation of other asexual spores like oidia, blastoconidia and arthroconidia has not been reported (Kües et al., 2016).

The cell wall of *S. commune* is essential for morphogenesis and for the interaction of this fungus with the (a)biotic environment. It consists of an inner, rigid core of α-(1,3)-glucan, β-(1,3)(1,6)-glucan (schizophyllan), chitin, polymeric fucose and polymeric mannose (Ehren et al., 2020; Safeer et al., 2023). The outer, mobile part of the cell wall is composed of β-(1,3)-glucan / β-(1,3)(1,6)-glucan (schizophyllan), α-(1,3)-glucan, polymeric mannose, protein, and can include polymeric (*N*-acetyl)galactosamine. In liquid shaking cultures, α-(1,3)-glucan is the most dominant polysaccharide in the inner, rigid part of the cell wall, while short β-glucan chains and schizophyllan are most dominant in the outer, mobile layer (Safeer et al., 2023). The β-(1,3)-glucans in the cell wall have a variety of functions. For instance, the β-(1,3)(1,6)-glucan schizophyllan protects against temperature cycles (Kleijburg et al., 2025). Moreover, it is thought to contribute to the tensile stiffness of the cell wall and to protect the mycelium from bacteria. Additionally, *S. commune* can use schizophyllan as a carbon source and can therefore be considered an extracellular storage sugar.

β-(1,3)-glucans are an integral cell wall component of many fungi. The synthesis of these cell wall polymers has been best studied in *Saccharomyces cerevisiae*. The membrane-bound β-(1,3)-glucan synthase complex consists of the catalytic subunit Fks and the regulatory subunit Rho1 (Li et al., 2025). The main part of Fks1 is localized in the cytosol, being anchored to the plasma membrane via 15 transmembrane (TM) helices that are clustered in two regions (TM domains) (Li et al., 2025). The cytosolic region encompasses the accessory (AC) and the glycosyltransferase (GT) domains. The GT domain belongs to the glycosyltransferase 48 family and synthesizes the β-(1,3)-glucan polymer. The AC domain interacts closely with the GT domain and is believed to have a regulatory function.

The *S. cerevisiae* genome contains the *FKS1* (SGD:S000004334), *FKS2* (also called *GSC2*; SGD:S000003264), and *FKS3* (SGD:S000004923) genes (Engel et al., 2025). *FKS2* is a paralog of *FKS1* that resulted from genome duplication (Kellis et al., 2004). *FKS1* is primarily expressed during vegetative growth on glucose, whereas *FKS2* is expressed during glucose starvation, sporulation, and in response to mating pheromones (Mazur et al., 1995). Deletion of *FKS1* or *FKS2* in *S. cerevisiae* is not lethal, but the combination of both is fatal (Douglas et al., 1994; Mazur et al., 1995). Deletion of *FKS1* results in a decrease in β-(1,3)-glucan in the cell wall and an increase in chitin, whereas deletion of *FKS2* results in defects in spore wall formation. *FKS3* expression is generally low (Ishihara et al., 2007) and can be deleted in a wildtype background or in combination with *FKS1 or FKS2.* Although the exact function of FKS3 is unclear, it is known to be involved in spore wall assembly like FKS2 (Ishihara et al., 2007).

β-(1,3)-glucan synthase (*bgs*) genes have also been identified in many other fungi. The ascomycete *Schizosaccharomyces pombe* contains four *bgs* genes (*bgs1–4*, Cortés et al., 2005), whereas the ascomycetes *Aspergillus nidulans*, *Aspergillus fumigatus*, *Neurospora crassa*, and *Fusarium solani* only contain a single *bgs* gene (*fksA*, *AfFks1, FKS,* and Fs*FKS1*, respectively; Kelly et al., 1996; Beauvais et al., 2001; Schimoler-O'Rourke et al., 2003; Ha et al., 2006). The mucoromycete *Rhizopus oryzae* also contains a single *bgs* gene (*FKS1*, Ibrahim et al., 2005), whereas *Mucor circinelloides* contains *fksA*, *fksB*, and *fksC* (Garcia et al., 2023). In basidiomycetes, *Malassezia sympodialis*, *Malassezia globosa*, *Ustilago maydis*, and *Cryptococcus neoformans* contain a single *bgs* gene, called *FKS1* (Gioti et al., 2013; *FKS1*, Thompson et al., 1999). By contrast, many mushroom-forming fungi contain two *bgs* genes; *Pholiota microspora* contains *PmBgs1* and *PmBgs2* (Zhu, 2018), *Grifola frondosa GFGLS* and *GFGLS2* (Jiang et al., 2022), *Pleurotus ostreatus fks1* and *fks2* (Schiphof et al., 2024), and *Ganoderma lucidum GLGLS1* and *GLGLS2* (Zhang et al., 2025). Phylogenetic analysis shows that the two *bgs* genes in Agaricomycetes encode a Type I and a Type II β-(1,3)-glucan synthase, with type-specific sequence signatures in part of the loop regions between the TM helices (Yang et al., 2014). Possibly, a gene duplication has occurred in the common ancestor of the Agaricomycetes, followed by divergent evolution resulting in the Type I and Type II β-(1,3)-glucan synthase genes (Yang et al., 2014). Whether these enzyme types have different functions is still unknown.

Here, we aimed to study the role of the Type I and Type II β-(1,3)-glucan synthase genes *bgs1* and *bgs2* of *S. commune*. The latter gene could not be inactivated, in contrast to *bgs1*. The Δ*bgs1* strain showed reduced growth, impaired fruiting body formation, and absence of spore production. In addition, this strain showed a changed cell wall composition and produced a lower amount of water-soluble polysaccharide with FTIR characteristics consistent with schizophyllan.

## 2 Materials and methods

### 2.1 Strains and culture conditions

The co-isogenic *S. commune* wild-type strains H4-8A (matA_43_matB_41_; FGSC no. 9210) (Ohm et al., 2010a) and H4-8B (matA_41_matB_43_) (Ohm et al., 2010b) and their derivatives H4-8AΔ*ku80* (de Jong et al., 2010), H4-8AΔ*bgs1,* H4-8BΔ*bgs1*, and H4-8BΔ*bgs1*::*bgs1* were grown at 30 °C in the dark on ammonium minimal medium (MM-N; Kleijburg et al., 2023) or *S. commune* minimal medium (SCMM; Dons et al., 1979). The media were prepared with or without 15 g l^−1^ agar, while SCMM agar was either supplemented or not with 15 μg ml^−1^ nourseothricin, 25 μg ml^−1^ phleomycin, or 20 μg ml^−1^ hygromycin (Bio-connect, www.bio-connect.nl).

Fruiting of *S. commune* dikaryons was induced by transferring 3-day-old, dark-grown colonies to the light and 25 °C, after which growth was prolonged for 7 days. To collect spores, plates were placed upside down for 1 day. Spores that had fallen on the Petri dish lid were collected in 1 ml Milli-Q water.

### 2.2 Bioinformatic comparison

The coding sequences of *bgs1* and *bgs2* were compared using the Utrecht University's Fungal Genomics Genome Portal (Beijen and Ohm, 2024; Marian et al., 2022). The GT domain was predicted using EMBL-EBI InterPro (Blum et al., 2025; Paysan-Lafosse et al., 2025), while TM helices were predicted with DTU Health Tech DeepTMHMM - 1.0 (Hallgren et al., 2022). Sequence motifs in the intrinsically disordered regions (IDRs) were analyzed using the Eukaryotic Linear Motif resource (Kumar et al., 2024). *S. commune* Bgs1 and Bgs2 were blasted in JGI's Mycocosm (Grigoriev et al., 2014) and a multiple sequence alignment was done on a selection of 38 Agaricomycete β-glucan synthases (Supplementary Table 1) using Clustal Omega Multiple Sequence Alignment (Madeira et al., 2024).

### 2.3 Inactivation of bgs1 of *S. commune*

The predicted *S. commune* β-(1-3)-glucan synthase gene bgs1 (Protein ID Schco3|2628724, Marian et al., 2022) was inactivated according to Vonk et al. (2019), while an attempt was made to inactivate bgs2 (Protein ID Schco3|2035630, Marian et al., 2022). To this end, pre-assembled Cas9-sgRNA ribonucleoprotein (RNP) complexes were used in combination with a repair plasmid in CRISPR-Cas9 mediated gene deletion. The Cas9 RNPs and the repair plasmids were introduced into *S. commune* protoplasts through PEG-mediated transformation. To construct the repair plasmids, an *Eco*RI fragment from pPV010 containing the nourseothricin-resistance cassette (Vonk and Ohm, 2021a) was placed in between the upstream and downstream sequences of the bgs1 and bgs2 open reading frames. In the case of bgs1, 1062 bp and 1069 bp flanking sequences were amplified from H4-8A genomic DNA with primers bgs1LeftFlank_fw/bgs1LeftFlank_rv and bgs1RightFlank_fw/bgs1RightFlank_rv, respectively (Table 1). In the case of bgs2, 1056 bp and 1051 bp flanking sequences were amplified with primers bgs2LeftFlank_fw/bgs2LeftFlank_rv and bgs2RightFlank_fw/bgs2RightFlank_rv, respectively. Next, the nourseothricin-resistance cassette, the PCR-amplified upstream and downstream flanks, and *Hin*dIII-digested pRO402 (consisting of a pUC19 backbone and a phleomycin-resistance cassette; Beijen et al., 2025) were assembled in a single step using the NEBuilder HiFi DNA Assembly Master Mix (New England Biolabs, international.neb.com). This resulted in deletion plasmids pESbgs1 and pESbgs2. The sgRNAs targeting bgs1 and bgs2 were synthesized in vitro from primer pairs bgs1sgRNA_left_fw/bgs1sgRNA_left_rv and bgs1sgRNA_right_fw/bgs1sgRNA_right_rv (*bgs1*) or bgs2sgRNA_left_fw/bgs2sgRNA_left_rv and bgs2sgRNA_right_fw/bgs2sgRNA_right_rv (*bgs2*) (Table 1) using the GeneArt Precision sgRNA Synthesis Kit (Thermo Fisher Scientific, www.thermofisher.com). The sgRNAs were pre-assembled with recombinant Cas9 protein into RNP complexes and introduced together with pESbgs1 and pESbgs2 into protoplasts of H4-8AΔ*ku80* using PEG mediated transformation (Vonk et al., 2019). Transformants resistant to nourseothricin but sensitive to phleomycin were predicted to have a *bgs1* or *bgs2* deletion. This was verified with PCR using primer pairs bgs1_ChkA/nour_ChkB and nour_ChkC/bgs1_ChkD or bgs2_ChkA/nour_ChkB and nour_ChkC/bgs2_ChkD, respectively (Table 1; Vonk and Ohm, 2021b). These primers anneal inside the nourseothricin-resistance cassette and upstream of the left flanking sequence or downstream of the right flanking sequence. Primer pairs bgs1_ChkA/bgs1_ChkB and bgs1_ChkC/bgs1_ChkD (Table 1) that anneal upstream of the left flanking sequence of *bgs1* and in the coding sequence of *bgs1*, and in the *bgs1* coding sequence and downstream of the right *bgs1* flank, respectively, were used as a negative control. Similarly, primer pairs bgs2_ChkA/bgs2_ChkB and bgs2_ChkC/bgs2_ChkD (Table 1) were used as a negative control for deletion of *bgs2*.

**Table 1:** Primers used in this study. Primers nour_ChkB and nour_ChkC are from Vonk and Ohm (2021b), while primers qPCR_arf1_fw / qPCR_arf1_rv have been published by d'Errico et al. (2025).

Name	Sequence
bgs1LeftFlank_fw	CTATGACCATGATTACGCCATGAAAGAAGGTGCTCCGG
bgs1LeftFlank_rv	GTCCCCCTCGAGGCGCGCCGGTGGTGATGGTTCGTGTTTG
bgs1RightFlank_fw	TCCCAGACCACCATGCCGGGTATCTAGACTTCTCCGCACC
bgs1RightFlank_rv	GATAACCTTCAGCAGAACGACTTGCGTCCTCGTACAAATC
bgs2LeftFlank_fw	CTATGACCATGATTACGCCATACTCGAGGCAGGGATACAG
bgs2LeftFlank_rv	GTCCCCCTCGAGGCGCGCCGCGTGCGTCGAAGAGTAGAG
bgs2RightFlank_fw	TCCCAGACCACCATGCCGGGACACACTGACATGGAATTGC
bgs2RightFlank_rv	GATAACCTTCAGCAGAACGATCGAGGACAGAGATAACGAC
bgs1sgRNA_left_fw	TAATACGACTCACTATAGGCTCCCACCACTACGATGCCG
bgs1sgRNA_left_rv	TTCTAGCTCTAAAACCGGCATCGTAGTGGTGGGAG
bgs1sgRNA_right_fw	TAATACGACTCACTATAGGATATTACAAGCTGTCGCAA
bgs1sgRNA_right_rv	TTCTAGCTCTAAAACTTGCGACAGCTTGTAATATC
bgs2sgRNA_left_fw	TAATACGACTCACTATAGTGTCCGGCCCAGGATATGGC
bgs2sgRNA_left_rv	TTCTAGCTCTAAAACGCCATATCCTGGGCCGGACA
bgs2sgRNA_right_fw	TAATACGACTCACTATAGGGAAACGTTAGTGAAAATAG
bgs2sgRNA_right_rv	TTCTAGCTCTAAAACCTATTTTCACTAACGTTTCC
bgs1_ChkA	CATTCCGACCAAAGCAAGAG
bgs1_ChkB	CATCGTAGTGGTGGGAGAAG
bgs1_ChkC	GCGACAGCTTGTAATATCGG
bgs1_ChkD	AGTCTCAGCACCGGGAATG
bgs2_ChkA	TCTGCAAGGAGATCTATGGC
bgs2_ChkB	TGGATTCCTGCCATATCCTG
bgs2_ChkC	GTCTATCGTATCGCCGGAC
bgs2_ChkD	TGGACACGAAGAGATCACTC
nour_ChkB	CCGAAAGATCCGATCGATAC
nour_ChkC	CGTCATGAATGAAGCCTCAG
bgs1_insert_fw	CATAAGCACCGAGGTTGCTCGGACTGGATATGGTCAGGCG
bgs1_insert_rv	CCCAGACCACCATGCCGGGCGAGGTTGCGGACAAGGTGTA
qPCR_bgs1_fw	GCGCCTATGATCCCTATCCG
qPCR_bgs1_rv	CGGAATGGCCAAACTTGTGG
qPCR_bgs2_fw	ACAGCGACAGAAACGGCAGC
qPCR_bgs2_rv	GAAGGGCGATATGCTGGGCA

### 2.4 Reintroduction of ku80 and complementation of ∆bgs1 of *S. commune*

To reintroduce *ku80*, a H4-8AΔ*ku80*Δ*bgs1* transformant was crossed with H4-8B wild type. Fructification was induced and spores were collected. Progeny was selected that was resistant to nourseothricin, indicative of *bgs1* deletion, but sensitive to hygromycin, indicative of the presence of *ku80* (de Jong et al., 2010). Deletion of *bgs1* in the F1 was confirmed with PCR using the primers as described above (data not shown).

To complement the Δbgs1 strain, a fragment encompassing the bgs1 promoter (1 to 890 bp upstream of the start codon), the bgs1 coding sequence including introns (6288 bp), and the bgs1 terminator (1 to 571 bp downstream of the stop codon) was introduced in Δbgs1 by random integration. The fragment was amplified from genomic DNA of H4-8A with primers bgs1_insert_fw / bgs1_insert_rv (Table 1). A two-fragment Gibson assembly was performed to insert the amplicon upstream of a nourseothricin-resistance cassette in *Nco*I digested pPV033, which also contains a phleomycin-resistance cassette (Vonk and Ohm, 2021a). The resulting complementation plasmid was introduced into protoplasts of H4-8BΔbgs1. Transformants resistant to phleomycin as well as to nourseothricin were predicted to have a randomly integrated bgs1 insert. This was verified with PCR using primers bgs1_insert_fw / bgs1_insert_rv (Table 1) that bind in the bgs1 promoter and terminator sequences, respectively, resulting in a 7460 bp fragment in a successful transformant (data not shown).

### 2.5 Expression of bgs1 and bgs2 in *S. commune* wild type, Δbgs1 and Δbgs1::bgs1

*S. commune* H4-8A, H4-8BΔ*bgs1*, and H4-8BΔ*bgs1*::*bgs1* were grown for 7 days on MM-N agar on a 76 mm polycarbonate track etch (PCTE) membrane (0.1 μm pore size, diameter 76 mm); GvS, www.gvs.com) sealed in a plastic bag (20 × 30 cm). RNA was isolated as described in d'Errico et al. (2025) and the expression of *bgs1* and *bgs2* was determined by qPCR using primer pairs qPCR_bgs1_fw / qPCR_bgs1_rv and qPCR_bgs2_fw / qPCR_bgs2_rv (Table 1), respectively. Expression of both genes was normalized to the expression of the constitutively expressed gene *arf1* using primer pair qPCR_arf1_fw / qPCR_arf1_rv (d'Errico et al., 2025).

### 2.6 Production of biomass and water-soluble polysaccharide

MM-N agar plates overlaid with a PCTE membrane were inoculated with an agar plug taken from the periphery of a 7-day old MM-N culture. Colonies were grown for 7 days sealed in a plastic bag (20 × 30 cm), after which they were harvested from the membrane, washed overnight in 10 ml dH_2_O at 20 rpm on a C1 Platform Shaker (New Brunswick Scientific, www.nbsc.com), rinsed twice with 10 ml dH_2_O, and freeze-dried. Polysaccharides were precipitated from the overnight washing solution by adding ethanol to a final concentration of 30% (*v*/v) and overnight incubation at 4 °C. The polysaccharides were harvested by centrifugation at 3200 *g* for 5 min at 4 °C and freeze-dried. The freeze dried fractions were analyzed by Fourier-transform infrared spectroscopy (FTIR) using a VerTex 70 FT-IR Spectrometer (Bruker, www.bruker.com) with a resolution of 2 cm^−1^ in the spectral range of 4000–550 cm^−1^.

The pH of the culture medium was measured after 7 days of growth with a LabArt series pH meter (Consort, www.consort.be). To this end, the agar underneath the colony was removed from the plate with a scalpel, pushed through a 5 ml syringe with a Luer-Lok tip (BD, www.bd.com) and collected in a 15 ml Greiner tube.

### 2.7 Spore counting

Dikaryons (*n* = 3 for each strain) were grown in the dark at high CO_2_ (9 cm plates sealed with parafilm) up to a diameter of 2 cm and transferred to generative conditions (low CO_2_, white light at 1500 lx). The plates were placed upside down when primordia had formed enabling spore harvesting from the lid of the plate using 500 μl water and a Drigalski spatula. This was done four days after mature fruiting bodies had developed. Dilutions of the spore suspensions resulting from the wildtype cross, the wildtype Δbgs1 cross and the Δbgs1 Δbgs1::bgs1 cross were used to count the basidiospores with a Bürker counting chamber. In the case of the Δ*bgs1*Δ*bgs1* strain, spores were counted after the 500 μl suspension was concentrated 25-fold by centrifugation for 5 min at 10,000 g followed by counting the whole sample without dilution. The same procedure was followed 7 and 14 days after mature fruiting bodies had formed, in case no spores were detected.

### 2.8 Cell wall composition

Petri-dish cultures (*n* = 1 for each strain), inoculated with an agar plug taken from the periphery of a 7-day-old MM-N agar grown colony, were grown sealed in a plastic bag (20 × 30 cm) for 7 days on a PCTE membrane on MM-N agar with ^13^C-glucose and ^15^NH_4_. The ^13^C- and ^15^NH_4_-labeled colony was harvested from the membrane and homogenized in an SK550 1.1 heavy-duty paint shaker (Fast & Fluid, www.fast-fluid.com) in a 15 ml Greiner tube for 9 min with five 4.76 mm (85 mg) metal beads (Bofix, www.bofix.nl) (hyphal fragmentation >95%). The cell walls were washed four times with 10 ml dH_2_O with centrifugation at 10,000 *g* for 10 min between the washes.

The water-washed cell walls were studied with fast magic angle spinning (MAS) solid state NMR (ssNMR) (Kleijburg et al., 2023; Safeer et al., 2023) using a narrow bore 16.4 T (700 MHz) NMR spectrometer with a 1.3 mm HXY MAS probe (Bruker BioSpin, www.bruker.com). Temperatures were set at 258 K at MAS frequencies of 54 kHz, which resulted in an actual sample temperature of 298 K due to frictional heating, as was previously shown by calibration with KBr powder (Thurber and Tycko, 2009). Rigid cell wall components were analyzed by dipolar-based ^1^H—^13^C 2D experiments using a ^13^C offset of 51 ppm with a spectral width of 130 ppm. 70–100% forward and backward cross-polarization (CP) ramps were used with 1.2 ms and 0.2 ms CP contact times, respectively, and PISARRO decoupling (Weingarth et al., 2008) was applied at 15 kHz. Flexible cell wall signals from polysaccharides and amino acid side chains were analyzed by scalar-based ^1^H—^13^C 2D experiments using a ^13^C offset of 57.3 ppm with a spectral width of 130 ppm, while WALTZ16 decoupling (Shaka et al., 1983) was performed at 10 kHz. For all experiments on the mobile cell wall 90° pulses were applied at 178 kHz for ^1^H and 93 kHz for ^13^C and max 200 ms of water suppression was achieved using MISSISSIPPI at 23 kHz (Zhou and Rienstra, 2008). ^1^H and ^13^C chemical shifts were referenced to the water peak at 4.7 ppm and to an external adamantane reference, respectively.

TopSpin 4.1 (Bruker BioSpin) was used to process and analyze NMR data. Peaks were integrated to determine the relative abundance analysis of rigid cell wall species in dipolar-based 2D ^1^H—^13^C spectra. Errors were based on the signal-to-noise of integrated peaks. For the relative abundance analysis of flexible polymer species in scalar-based 2D ^1^H—^13^C spectra, cumulative peak intensities per cell wall polymer were considered (Safeer et al., 2023) using only the representative polysaccharide peaks in the carbohydrate C1 region (^13^C dimension: 90–110 ppm range). Errors were based on the signal-to-noise of peak intensities.

The standard deviation (SD) (EQ.1) and standard error (SE) (EQ.2) were calculated from the spectral integration values used to determine the relative abundances of components in the rigid and mobile domains.

(1) $$\mathrm{SD}=\sqrt{\left(\sum_{i=1}^{N}{\left(x_{i}-\mu \right)}^{2}\right)/N}$$

with x_i_ = Individual component; μ = mean of the components; N = total numbers of the components

(2) $$\mathrm{SE}=\frac{\mathrm{SD}}{\sqrt{N}}$$

With N = total number of the components.

### 2.9 Statistics

Statistical analysis of biomass, schizophyllan production, and pH of the culture media was done using *t*-tests (*p* ≤ 0.05) with a Shapiro Wilk's test to confirm normality and a Levene's test to confirm equal variance.

## 3 Results

### 3.1 bgs1 and bgs2 of *S. commune*

Bidirectional blasting of the β-(1,3)-glucan synthase genes FKS1, FKS2, and FKS3 of *S. cerevisiae* against the genome of *S. commune* (Marian et al., 2022) revealed the predicted orthologues bgs1 (Schco3|2628724) and bgs2 (Schco3|2035630). The *S. commune* genes share 48.8–55.0% identity with their yeast counterparts (Table 2). The lowest shared identity was between bgs1 and FKS3 (48.8%) and bgs2 and FKS3 (49.4%). Genes bgs1 and bgs2 of *S. commune* are 71.3% identical and share 83.4% similarity. These genes are predicted to encode Type I and Type II agaricomycete β-glucan synthases, respectively (Yang et al., 2014). The 1784 and 1741 amino acid proteins consist of a GT domain, 17 predicted TM helices and an intrinsically disordered region (IDR) at the start of the polypeptide (amino acids 1–121 and 1–91 in BGS1 and BGS2, respectively) (Supplementary Fig. 2). There is a clear distinction between the IDRs of Type I and Type II β-glucan synthases. These synthases contain a DPF and NPF motif, respectively (Supplementary Fig. 2), that function in clathrin-mediated endocytosis by binding to different proteins in this pathway (Azarnia Tehran et al., 2019).

**Table 2:** Amino acid homology between the predicted β-(1,3)-glucan synthase genes *bgs1* and *bgs2* of *S. commune* H4-8 and the FKS1, FKS2, and FKS3 genes of *S. cerevisiae* S288C. Pairwise amino acid sequence identity was determined using NCBI BLASTP based on alignment of full-length predicted protein sequences. Percentage identity was calculated as the number of identical residues divided by the total alignment length. Similarity was calculated as the percentage of aligned residues that were either identical or represented conservative substitutions.

	*S. cerevisiae* protein									*S. commune* protein		
	FKS1			FKS2			FKS3			Bgs2		
*S. commune* protein	Query coverage	Identity	Similarity	Query coverage	Identity	Similarity	Query coverage	Identity	Similarity	Query coverage	Identity	Similarity
Bgs1	100%	55.0%	70.8%	99%	54.8%	70.2%	92%	48.8%	66.5%	98%	72.0%	84.1%
Bgs2	98%	54.0%	70.4%	99%	53.9%	70.1%	93%	49.4%	66.1%			

Data from Krizsán et al. (2019) show that bgs1 and bgs2 of *S. commune* have a similar expression pattern during development of fruiting bodies (Table 3). Expression of both genes is significantly increased in stage II primordia compared to vegetative mycelium, stage I primordia, young fruiting bodies, and mature fruiting bodies.

**Table 3:** Expression (Fragments Per Kilobase Million) of *bgs1* and *bgs2* in *S. commune* H4-8 in vegetative mycelium (VM), stage I primordia (P1), stage II primordia (P2), young fruiting bodies (YFB), and mature fruiting bodies (FB) when grown on SCMM with glucose (data from Krizsán et al., 2019). Different letters in parentheses indicate statistical differences in expression between the different developmental stages. For instance, expression of *bgs1* in VM is not different when compared to P1 but is different when compared to P2. Similarly, expression of *bgs2* is not different from any of the other stages.

gene	Expression during fruiting body development (FPKM)				
	VM	P1	P2	YFB	FB
*bgs1*	59.3 ± 22.9 (a)	95.6 ± 59.7 (a)	187.8 ± 179.2 (b)	66.9 ± 37.8 (a)	70.1 ± 46.2 (a)
*bgs2*	104.5 ± 50.3 (ab)	71.1 ± 43.4 (a)	209.3 ± 182.6 (b)	77.9 ± 47.1 (a)	58.3 ± 37.5 (a)

### 3.2 Deletion of bgs1

Deletion constructs for bgs1 and bgs2 were introduced in *S. commune*. A Δbgs1 strain was successfully obtained. The replacement of the bgs1 coding sequence by a nourseothricin-resistance cassette was confirmed with PCR. However, despite five attempts, a bgs2 deletion transformant was not obtained in H4-8AΔku80 using Cas9-mediated transformation. Instead of replacing bgs2 via a double cross-over, 15 transformants integrated the repair plasmid via a single cross-over only (data not shown).

The H4-8AΔku80Δbgs1 deletion strain was crossed with H4-8B to reintroduce ku80. The resulting spores were collected and individually screened for nourseothricin resistance (indicative of the absence of bgs1) and hygromycin sensitivity (indicative of the presence of ku80). This resulted in the compatible H4-8AΔbgs1 and the H4-8BΔbgs1 deletion strains, as was confirmed with PCR (data not shown) and qPCR (Supplementary Table 2). Next, the deletion strain H4-8BΔbgs1 was complemented with an intact copy of bgs1. Insertion of the gene was confirmed through PCR (data not shown). In addition, qPCR showed restored expression of bgs1 in the H4-8BΔbgs1::bgs1 strain (Supplementary Table 2). Of interest, qPCR also demonstrated that inactivation of bgs1 did not increase expression of bgs2 in the deletion strain.

### 3.3 Production of biomass and water-soluble polysaccharide

The co-isogenic *S. commune* wild type H4-8A and the H4-8BΔbgs1 and H4-8BΔbgs1::bgs1 strains were grown on a PCTE membrane overlaying MM-N agar. Colonies of Δbgs1 were smaller and more dense when compared to the wild type and Δbgs1::bgs1 (Fig. 1). The colonies were washed with water, after which mycelium dry weight was determined and polysaccharide was precipitated from the wash water with 30% ethanol. The Δbgs1 strain produced less biomass (0.03 g) than the wild type (0.06 g) and Δbgs1::bgs1 (0.06 g) strains (Table 4). The ethanol precipitates of the wild-type, Δbgs1, and Δbgs1::bgs1 showed similar FTIR spectra (Supplemental Fig. 1) resembling that of purified schizophyllan (Kleijburg et al., 2025). The Δbgs1 strain produced less ethanol precipitate (27.9 mg g^−1^ mycelium) than the wild type (54.71 mg g^−1^ mycelium) and Δbgs1::bgs1 (44.34 mg g^−1^ mycelium) (Table 4).

**Fig. 1:**
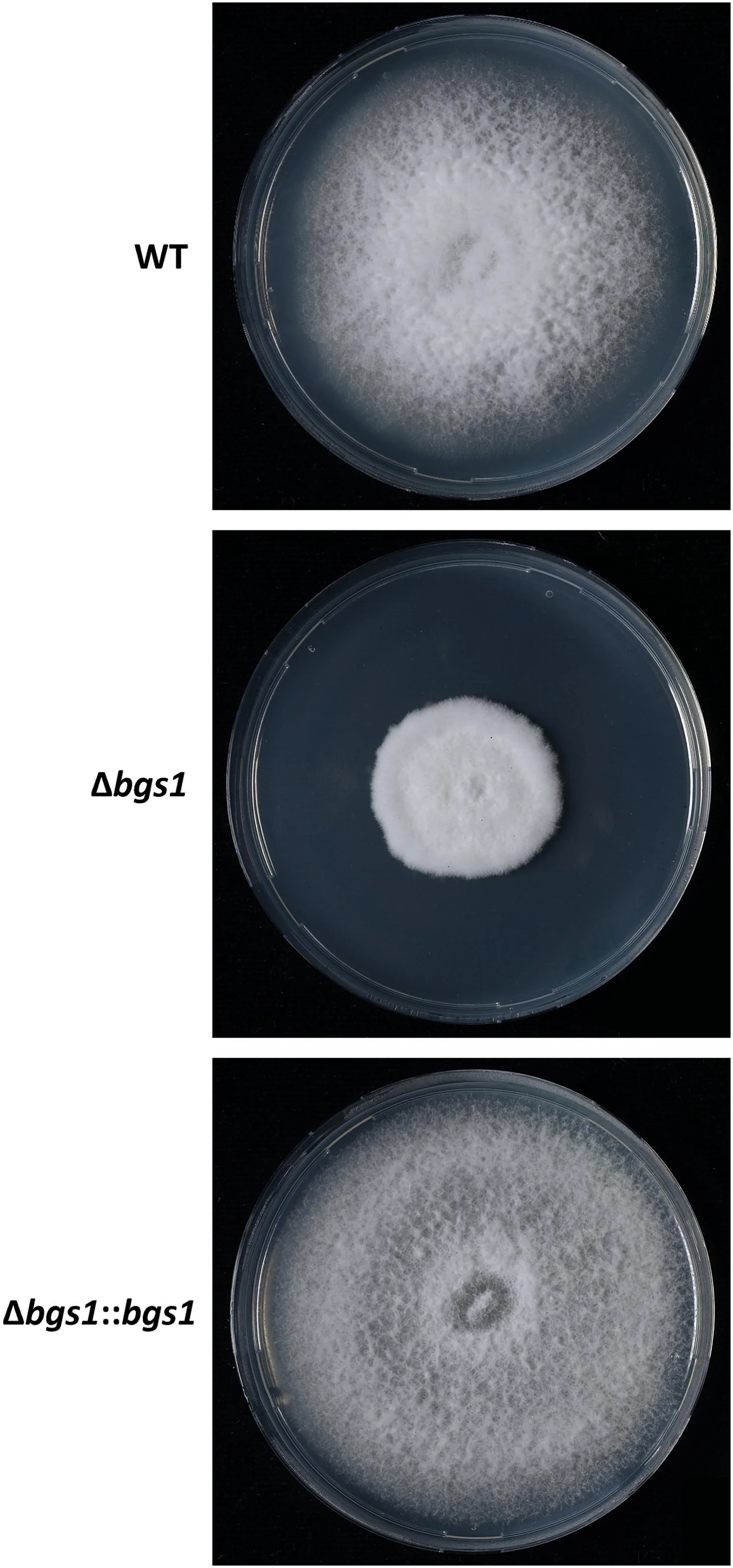
Morphology of the co-isogenic *S. commune* strains wild type H4-8A, H4-8BΔ*bgs1*, and H4-8BΔ*bgs1*::*bgs1* grown on MM-N agar (*n* = 3).

**Table 4:** Production of dry weight biomass and water-soluble polysaccharide (WSP) with FTIR characteristics consistent with schizophyllan as well as pH of the culture medium of 7-day-old MM-N agar cultures of the co-isogenic strains *S. commune* wild type H4-8A, H4-8BΔ*bgs1,* and H4-8BΔ*bgs1*::*bgs1*. Values are mean ± SD. Significant differences (*p* ≤ 0.05) within a column are indicated with different letters. For instance, mass of mycelium of H4-8A is different from H4-8BΔ*bgs1,* but not different from H4-8BΔ*bgs1*::*bgs1.* Similarly, WSP per mg biomass of H4-8BΔ*bgs1*::*bgs1* is not different from that of H4-8A and H4-8BΔ*bgs1.*

Strain	Mycelium (g)	WSP (mg)	WSP (mg g^−1^ biomass)	pH
H4-8A WT	0.06 ± 0.01 (a)	3.16 ± 0.35 (a)	54.7 ± 16.9 (a)	2.71 ± 0.02 (a)
H4-8BΔ*bgs1*	0.03 ± 0.01 (b)	0.90 ± 0.17 (b)	27.9 ± 8.74 (b)	3.52 ± 0.11 (b)
H4-8BΔ*bgs1*::*bgs1*	0.06 ± 0.01 (a)	2.83 ± 0.58 (a)	44.3 ± 5.59 (ab)	2.85 ± 0.20 (a)

Previously, we showed that *S. commune* secretes more schizophyllan when the pH of the culture medium is ≤5.2 (Kleijburg et al., 2025). The pH of the medium was lower than this pH for all three strains (ranging from 2.7 to 3.5; Table 4). Together, results show that deletion of bgs1 impacts the production of biomass and water-soluble polysaccharide with FTIR characteristics consistent with schizophyllan.

### 3.4 Formation of fruiting bodies and basidiospores

Because *S. commune* does not fructify on MM-N agar, SCMM agar was used. Wild type H4-8A and deletion strain H4-8AΔbgs1 were crossed with either the co-isogenic strains H4-8B wild type, H4-8BΔbgs1, or H4-8B Δbgs1::bgs1. The Δbgs1Δbgs1 dikaryon formed smaller fruiting bodies than the wild type (Fig. 2, Fig. 3). However, the hymenium of wild type fruiting bodies had a ribbed surface, whereas a smooth surface was observed in the Δbgs1Δbgs1 fruiting bodies (Fig. 4AB). Microscopic assessment of the hymenium showed the formation of basidia in fruiting bodies of all strains (Fig. 4C-F). However, the Δbgs1Δbgs1 basidia did not produce any spores, while the wildtype cross, the wildtype Δbgs1 cross and the Δbgs1 Δbgs1::bgs1 cross formed 129.8 10^6^ ± 16.3 10^6^, 55 10^6^ ± 14.4 10^6^ and 14.5 10^6^ ± 3.2 10^6^ spores, respectively. In addition, the Δbgs1Δbgs1 fruiting bodies were less firm (based on qualitative assessment) and released a yellow/orange exudate, that surrounded the fruiting bodies (Fig. 3B). Moreover, the Δbgs1Δbgs1 colonies formed a blue pigment (Fig. 2C), accompanied by a dark discoloration of both the colony and the agar medium (Fig. 2B). Crossing the Δbgs1 strains with a compatible wild type strain or the compatible Δbgs1::bgs1 strain showed normal growth and fruiting body formation (Fig. 2, Fig. 3). Together, a single bgs1 copy in the dikaryon is sufficient for full development but not for the production of the same number of basidiospores when compared to a wild type bgs1bgs1 dikaryon.

**Fig. 2:**
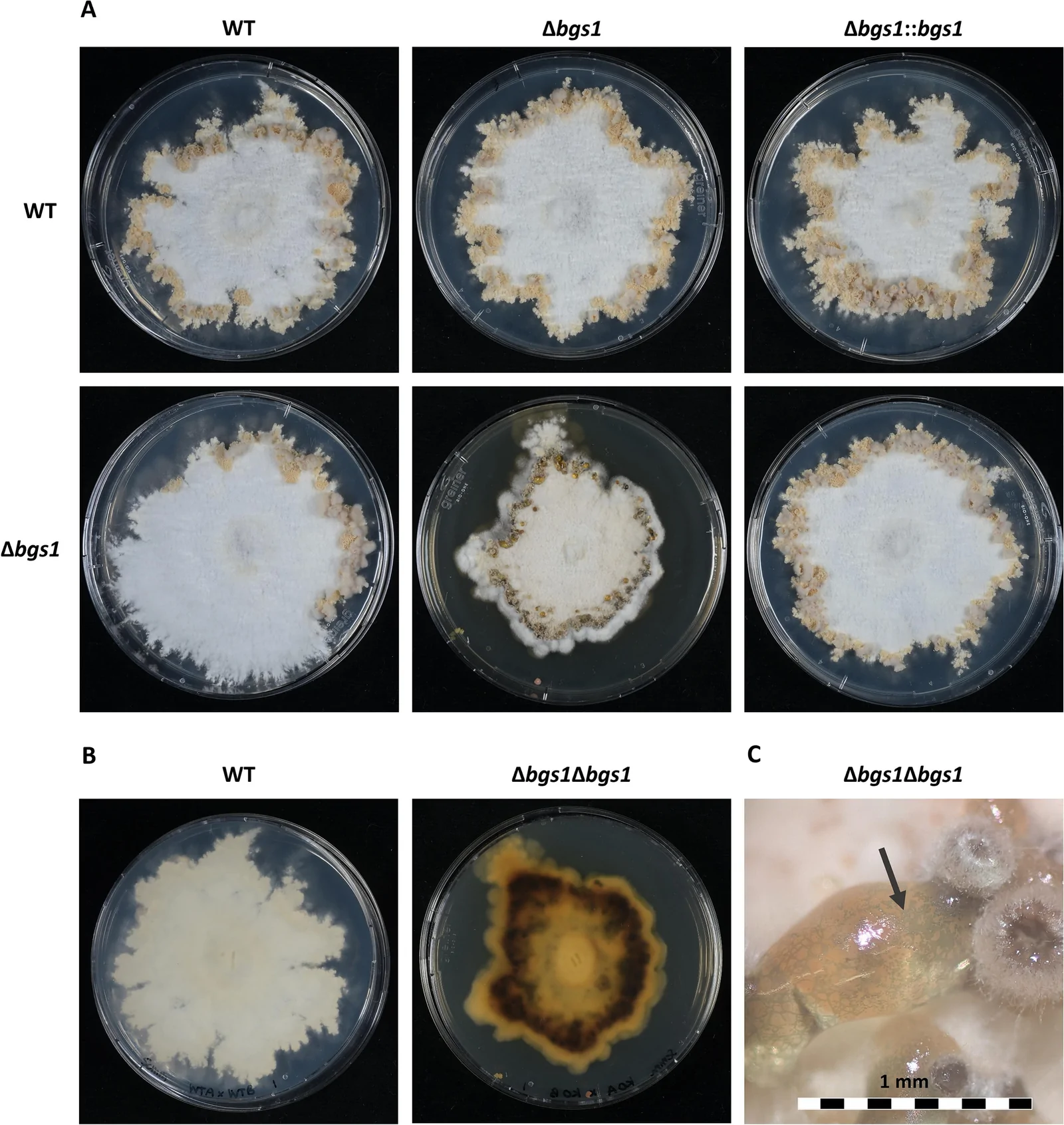
Top (A) and bottom (B) view of crossings of compatible combinations of co-isogenic *S. commune* wild type, Δ*bgs1*, and Δ*bgs1*::*bgs1* strains (*n* = 3). The Δ*bgs1*Δ*bgs1* crossing produced smaller colonies and fruiting bodies (A), and a dark discoloration of the colony (B), including the production of blue pigment (C). Bar represents 1 mm, while arrow indicates the blue pigment that is released into the excreted droplets. Normal fruiting bodies are produced when at least one of the mating partners has an intact *bgs1* gene (i.e. a wild type or a complemented strain). (For interpretation of the references to colour in this figure legend, the reader is referred to the web version of this article.)

**Fig. 3:**
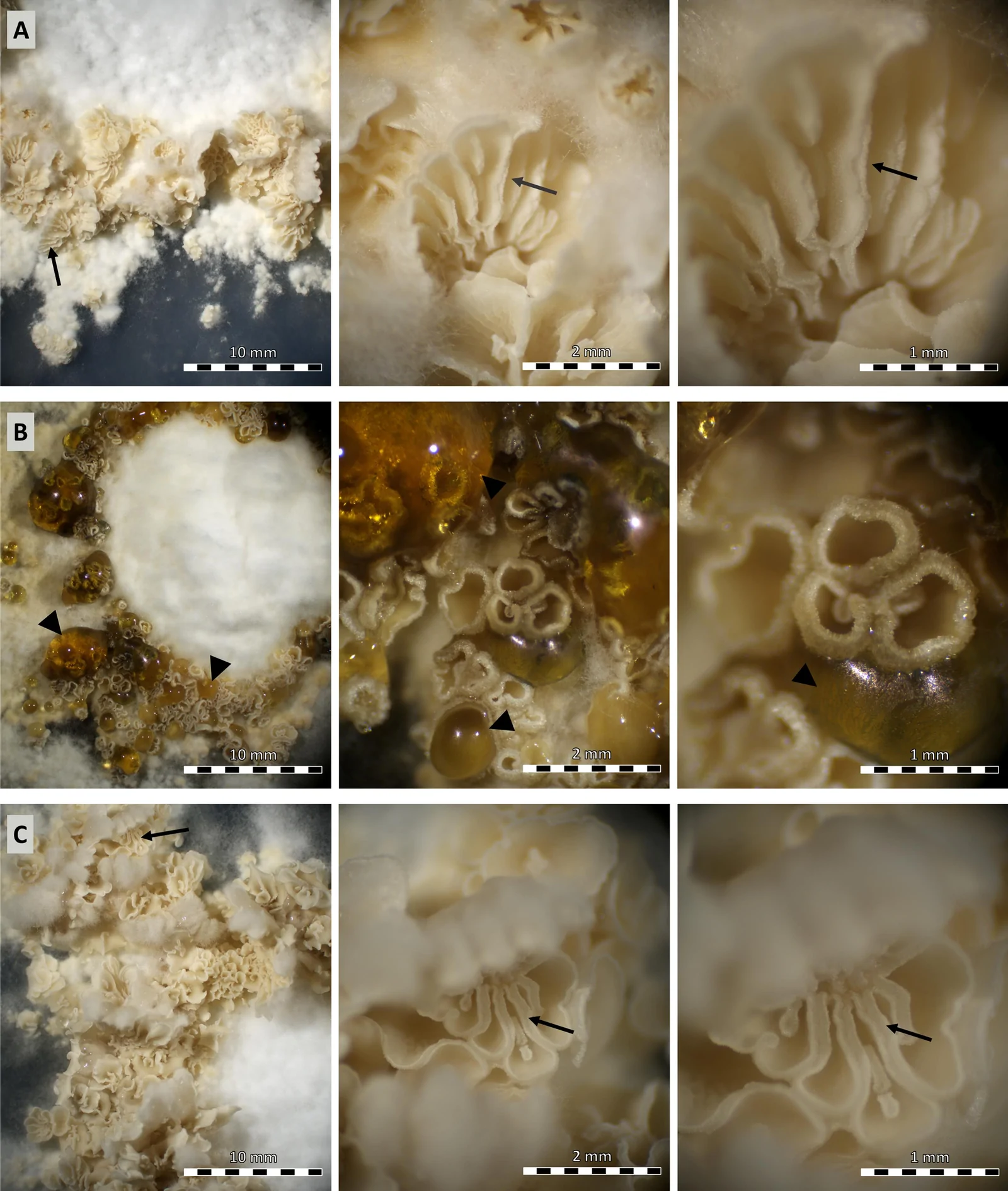
Fruiting body formation in the co-isogenic *S. commune* strains wild type H4-8AB (A), H4-8ABΔ*bgs1*Δ*bgs1* (B), and H4-8AΔ*bgs1* x H4-8BΔ*bgs1::bgs1* (C) (*n* = 3). Arrows indicate the split gills. The Δ*bgs1*Δ*bgs1* strain produces smaller fruiting bodies that excrete yellow/orange droplets (indicated by arrow heads). (For interpretation of the references to colour in this figure legend, the reader is referred to the web version of this article.)

**Fig. 4:**
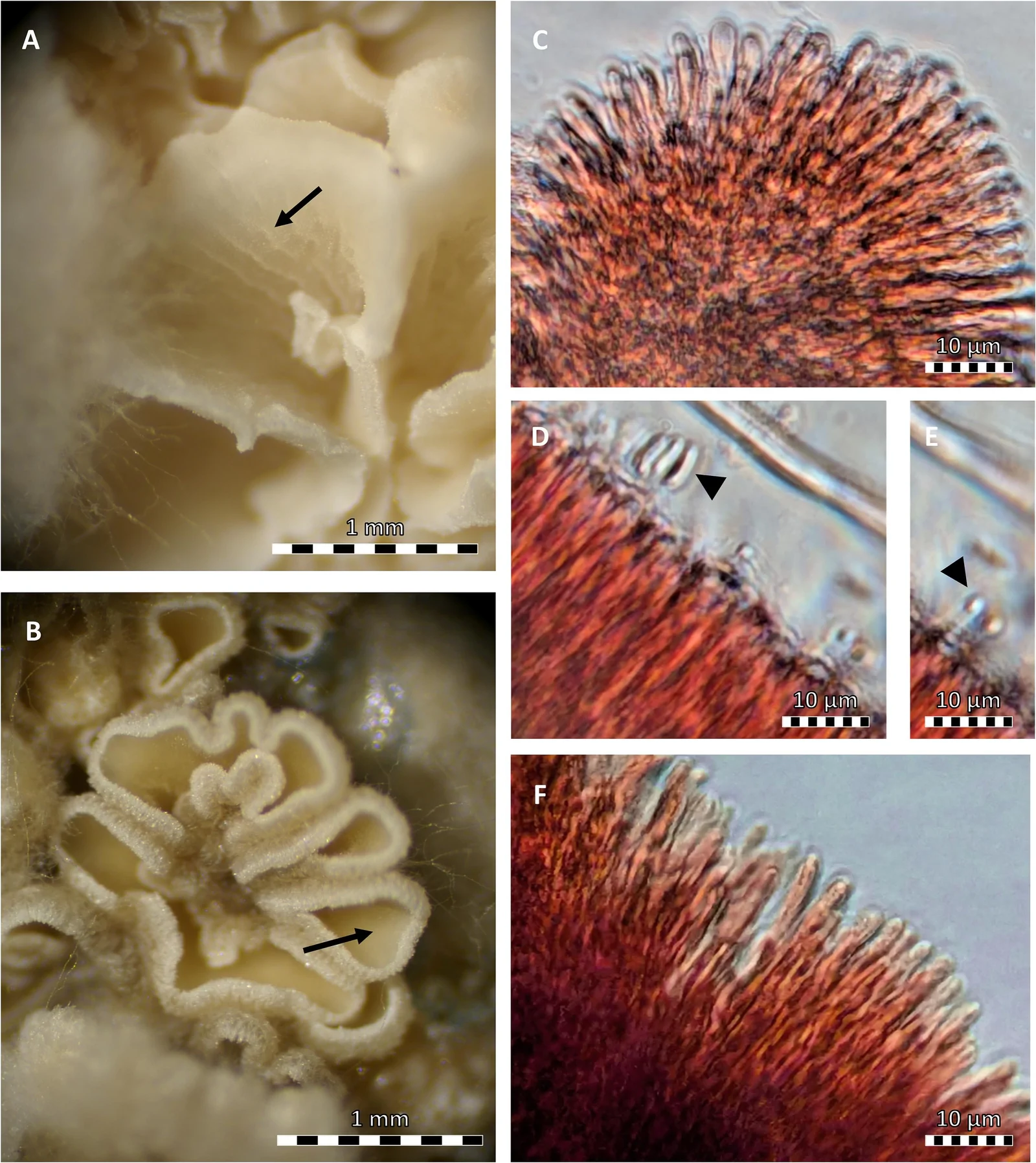
Hymenia (A,B) and basidia (C–F) of *S. commune* wild type H4-8AB (A, C–E) and H4-8ABΔ*bgs1*Δ*bgs1* (B, F) (*n* = 3). Arrows indicate a ribbed hymenium in wild type fruiting bodies (A), whereas the hymenium is smooth in the case of the Δ*bgs1*Δ*bgs1* strain (B). The wild-type hymenium forms basidia (C-E) that form basidiospores (indicated by arrow heads) (D,E), while the basidia of the Δ*bgs1*Δ*bgs1* strain do not form spores (F). D and E show mature and developing spores, respectively.

### 3.5 Cell wall composition

The composition of water-extracted cell walls of the H4-8BΔbgs1 strain was compared to that of the co-isogenic wild type strain H4-8A with solid-state NMR. To this end, the strains were grown on MM-N agar with ^13^C-glucose and ^15^NH_4_. The mobile part of the cell wall of the Δbgs1 strain contained relatively 47% β-(1,3)-glucan / β-(1,3)-(1,6)-glucan, which was lower than the wild-type that contained relatively 58% of this polysaccharide. The mobile part of the cell wall of the Δbgs1 strain also contained relatively lower levels of α-(1,3)-glucan when compared to the wild-type (16% and 28%, respectively) (Fig. 5). On the other hand, lipids (15%) and amino acids (17%) were higher in the cell wall preparation of the Δbgs1 strain when compared to the wild-type strain (15% and 3% and 17% and 6%, respectively). The lipids, and therefore part of the proteins, are expected to originate from the plasma membrane (Safeer et al., 2023). Consequently, the mobile part of the cell wall of the wild-type and Δbgs1 strains are assumed to be similar. Relative differences in the rigid part of the cell wall of the Δbgs1 and wild-type strains were pronounced. A relative level of 14% β-(1,3)-glucan / β-(1,3)(1,6)-glucan was detected in the rigid cell wall of the Δbgs1 strain, while this was 70% in the wild-type. By contrast, the deletion strain contained relative levels of 51% α-(1,3)-glucan and 30% chitin, while these relative levels were only 19% and 9% in the wild-type, respectively. The spectra of the wild type and the Δbgs1::bgs1 strain were similar (Supplementary Fig. 3), implying that the cell wall composition was restored after complementation of the Δbgs1 strain. Together, inactivation of bgs1 reduced the relative amount of β-(1,3)-glucan / β-(1,3)(1,6)-glucan in the rigid core of the cell wall, which was accompanied by higher relative levels of α-(1,3)-glucan and chitin.

**Fig. 5:**
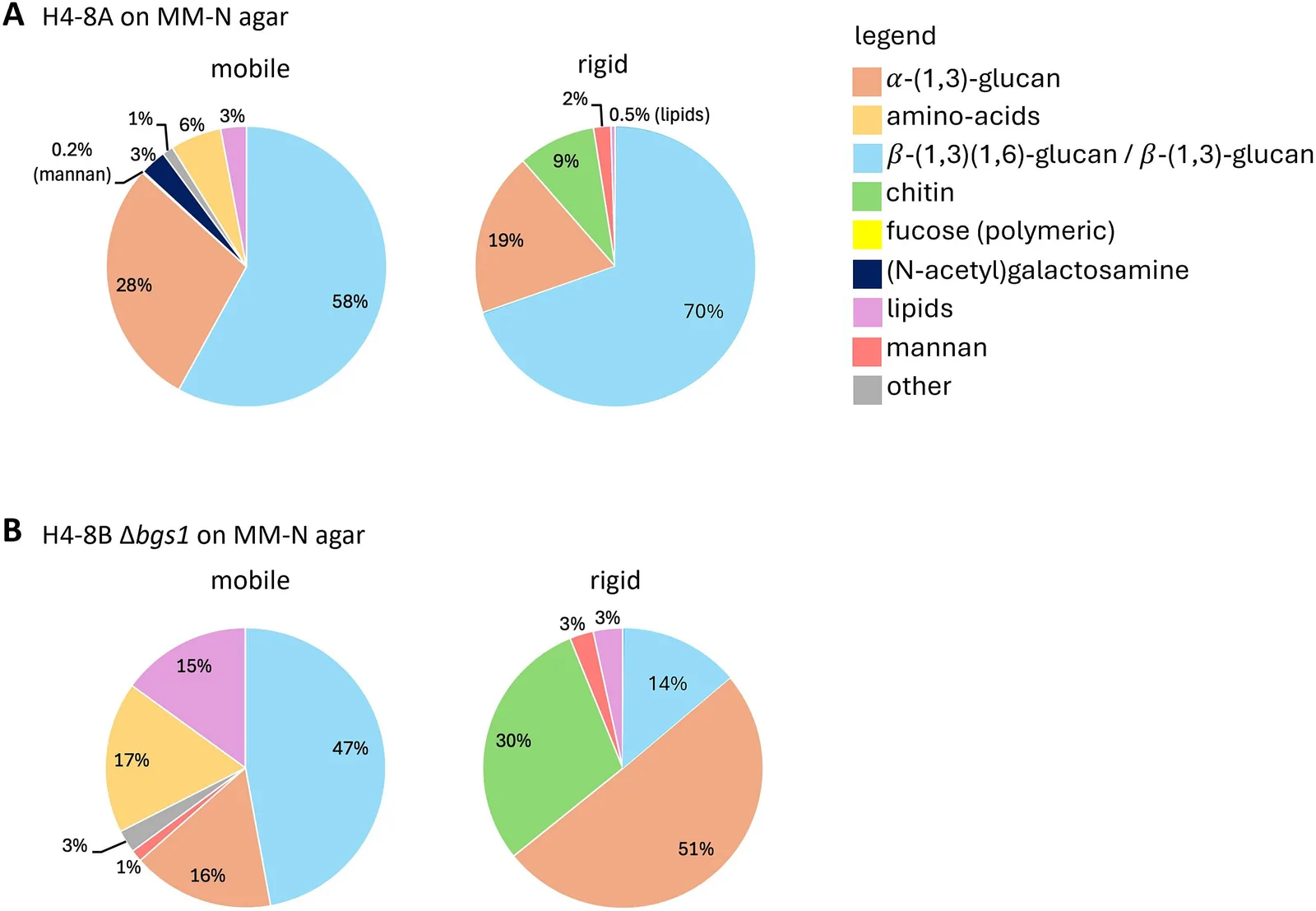
Quantitative assessment of the mobile and rigid cell wall of the co-isogenic wild type (SD 20.0 and SE 7.6 for the mobile phase and SD 25.8 and SE 11.3 for the rigid phase) and Δ*bgs1* (SD 15.1 and SE 5.7 for the mobile phase and SD 18.3 and SE 8.2 for the rigid phase) strains grown on MM-N agar (*n* = 1 for each strain). The spectra of the mobile and rigid cell wall of the complemented strain Δ*bgs1::bgs1* are similar to that of the wild type strain (Supplementary Fig. 2). SD and SE were calculated from the spectral integration values used to determine the relative abundances of components in the rigid and mobile cell wall domains.

## 4 Discussion

So far, the role of β-(1,3)-glucan synthase genes in mushroom-forming fungi has only been studied in *Grifola frondosa*. A glucan synthase gene, *GFGLS,* was silenced by RNAi to 26% of that found in the wild-type (Cui et al., 2019). This resulted in a 29% reduction of colony extension when grown on agar medium and a 80% reduction in biomass in liquid shaken cultures. Qualitative assessment showed that hyphae of the silenced strain were thinner and showed a reduced branching incidence. Also, exopolysaccharide production was about 5-fold reduced per mycelium biomass when compared to the wildtype. Here, we aimed to study the role of the predicted β-(1,3)-glucan synthase genes *bgs1* and *bgs2* of *S. commune*. However, a deletion strain was only obtained for *bgs1.* Like *GFGLS* of *G. frondosa*, *bgs1* was shown to be involved in vegetative growth and release of extracellular polysaccharides. Apart from this, *bgs1* was shown to be involved in cell wall composition, fruiting body formation and basidiospore development.

Genes *bgs1* and *bgs2* were identified by bidirectional blasting against the *FKS1-3* genes of *S. cerevisiae.* These genes shared the highest identity with *FKS1* (55.0% and 54.3%, respectively) and FKS2 (54.9% and 54.8%, respectively). The encoded proteins are predicted to be Type I and Type II Agaricomycete β-(1-3)-glucan synthases (Yang et al., 2014), each containing 17 predicted TM α-helices. FKS1 of *S. cerevisiae* was previously also predicted to contain this number of helices (Hu et al., 2023) but recent cryo-EM analysis revealed that FKS1 only contains 15 TM helices (Li et al., 2025), demonstrating that the computational prediction may overestimate TM numbers in glucan synthases, including those of *S. commune*. In addition, Bgs1 and Bgs2 of *S. commune* are predicted to have an intrinsically disordered region (IDR) at the N-terminus of the polypeptide. IDRs play a role in molecular recognition and signaling processes. Interestingly, the evolutionary divergence between the Type I and Type II β-(1,3)-glucan synthases in Agaricomycetes (Yang et al., 2014) is also reflected in these IDRs. Type I (Bgs1-like) and Type II (Bgs2-like) β-(1,3)-glucan synthases contain a DPF and a NPF motif, respectively. These motifs function in clathrin-mediated endocytosis (Azarnia Tehran et al., 2019). NPF motifs mediate interactions with EH (Eps15 Homology) domains, which are found in proteins involved in intracellular membrane trafficking and endocytosis (Salcini et al., 1997; de Beer et al., 1998). The DPF motif binds to the adaptor protein 2 (AP2) complex, which facilitate the assembly of clathrin lattices during clathrin-mediated endocytosis (Owen et al., 1999; Brett et al., 2002). Future studies should assess whether the NPF and DPF motifs have functional consequences for the Type I and Type II β-(1,3)-glucan synthases of *S. commune*.

Inactivation of *bgs1* resulted in a 2-fold lower biomass after 7 days of growth. This may be explained by the changed cell wall composition of the deletion strain. In particular, a 5-fold reduction in the relative abundance of β-(1,3)-glucan / β-(1,3)(1,6)-glucan was found in the rigid part of the cell wall, which may be compensated (in part) by the relative increase in α-(1,3)-glucan (168%) and chitin (233%). However, this compensation should be confirmed in future studies since our NMR data revealed relative but not absolute levels of the cell wall polymers. Yet, decreased levels of β-(1,3)-glucan and increased levels of chitin in the cell wall were also observed upon deletion of *fks1* in *S. cerevisiae* (Ram et al., 1998). Notably, highly decreased relative levels of schizophyllan (in fact, it was not detected at all) and increased relative amounts of α-(1,3)-glucan and chitin were also observed when wildtype *S. commune* was grown on wood (Kleijburg, 2025). This is interesting in light of the finding that such a change in cell wall composition (obtained by adding 10-fold KH_2_PO_4_/K_2_HPO_4_ buffer to keep the pH of the medium at 6.4; see below) results in a less strong but more ductile mycelium (with a higher elongation at break; Kleijburg et al., 2025). Together, we hypothesize that *S. commune* modulates levels of β-(1-3)-glucans, α-(1,3)-glucan, and chitin to adapt its cell wall composition to enable growth in different substrates.

Addition of 10-fold KH_2_PO_4_/K_2_HPO_4_ buffer to MM-N (MM-NKP) was found to decrease the production of biomass by the *S. commune* wild-type by 50% (Kleijburg et al., 2025). In addition, a 4.2-fold reduction in release of β-(1,3)(1,6)-glucan in the medium was observed per g biomass. Inactivation of bgs1 had a similar effect on production of biomass and water-soluble polysaccharide with a FTIR spectrum consistent with schizophyllan; both were decreased 2-fold. It has been shown that *S. commune* secretes more schizophyllan when the pH of the culture medium is ≤5.2 (Kleijburg et al., 2025). The pH of the medium in this study ranged between 2.7 (wild-type) and 3.5 (Δbgs1). This pH difference may explain (in part) why the wildtype secretes more water-soluble polysaccharide (presumably schizophyllan) than Δbgs1. It seems that deletion of *bgs1* and growth on MM-NKP have additive (or synergistic) effects on biomass formation and the release of water-soluble schizophyllan; the Δbgs1 strain hardly grew on MM-NKP and no water-soluble schizophyllan was produced (Kleijburg, 2025). A similar effect seems to hold for schizophyllan in the rigid part of the cell wall. The rigid cell wall of Δ*bgs1* consisted of relatively 80% less β-(1,3)(1,6)-glucan, while growth of the wild-type on MM-NKP reduces the relative levels of β-(1,3)(1,6)-glucan in the rigid cell wall by 89% (Kleijburg et al., 2025). Notably, not only production of biomass and water-soluble polysaccharide (presumably schizophyllan) were similarly affected when bgs1 was inactivated or when the wildtype strain was grown on MM-NKP medium, also the ability to lift the mycelium from the agar medium was identical. Unlike the mycelium of wild-type grown on MM-N agar, the mycelium of Δ*bgs1* grown on MM-N or wild type mycelium grown on MM-NKP agar could be easily lifted from the agar medium (Kleijburg, 2025). Kleijburg et al. (2025) suggested that the lifting phenotype was due to the absence of (*N*-acetyl)galactosamine in the outer cell wall of wild type mycelium growing on MM-NKP agar. This hypothesis is strengthened by the fact that the mobile cell wall of Δ*bgs1* also lacks polymers containing (*N*-acetyl)galactosamine.

Inactivation of bgs1 also resulted in impaired fruiting body development. The Δbgs1 deletion strain produced smaller fruiting bodies compared to the wild type. In addition, the Δbgs1 fruiting bodies had a softer texture and reduced firmness (based on qualitative assessment), excreted yellow/orange droplets, and did not produce spores. We hypothesize that the reduced firmness of the fruiting bodies and excretion of droplets by these reproductive structures is caused by a weaker cell wall that can no longer maintain the osmotic pressure. Indeed, inhibition of β-(1,3)-glucan synthesis, e.g. by echinocandin antifungals, weakens the fungal cell wall (Walker and Munro, 2020). As a result, the weaker cell wall has a reduced ability to withstand osmotic pressure, making the cell more susceptible to leakage (Safdar, 2009). Moreover, a reduced ability to withstand turgor pressure could reduce swelling of the fruiting bodies, thereby explaining the reduced size of these reproductive structures. Of interest, bgs1 and bgs2 are more highly expressed in the stage II primordia when compared to the other developmental stages (Krizsán et al., 2019). This suggests that β-(1,3)-glucan synthesis is even more important in these primordia when compared to the other developmental stages. Consequently, inactivation of bgs1 would have a stronger effect in this developmental stage than for instance in the vegetative mycelium. We hypothesize that this explains the leakage of droplets from fruiting bodies, while this is not observed in the vegetative mycelium. A high osmotic pressure may also be required for the formation of basidiospores by basidia. Therefore, we hypothesize that the lack of basidiospore formation in the Δbgs1 deletion strain is explained by the absence of a high osmotic pressure.

Together, this study has shown for the first time the impact of deletion of a β-(1,3)-glucan synthase gene on cell wall composition, growth, and fruiting body development in a mushroom-forming fungus. Future studies should show whether bgs2 is essential, which would explain why we were not able to inactivate this gene in *S. commune*.

## CRediT authorship contribution statement

**Ella M. Schunselaar:** Writing – review & editing, Writing – original draft, Methodology, Investigation, Conceptualization. **Fleur E.L. Kleijburg:** Writing – review & editing, Methodology, Investigation, Conceptualization. **Adil A. Safeer:** Writing – review & editing, Writing – original draft, Methodology, Investigation, Conceptualization. **Ajit K. Bishoyi:** Writing – review & editing, Investigation. **Sophie J.L. van Velzen:** Methodology, Investigation. **Peter Jan Vonk:** Writing – review & editing, Supervision, Methodology, Investigation. **Luis G. Lugones:** Writing – review & editing, Supervision, Methodology, Investigation, Conceptualization. **Marc Baldus:** Writing – review & editing, Supervision, Funding acquisition. **Han A.B. Wösten:** Writing – review & editing, Supervision, Funding acquisition, Conceptualization.

## Funding

This work was funded by the Dutch Research Council NWO domain Applied and Engineering Sciences (grant number 18425), the Fungateria Horizon-EIC-2021-Pathfinder Challenges project (Grant agreement 101071145) as well as the National Roadmap Large-Scale NMR Facility of the Netherlands (uNMR-NL grid, grant number 184.035.002).

## Declaration of competing interest

The authors declare that they have no known competing financial interests or personal relationships that could have appeared to influence the work reported in this paper.

## Data Availability

Data will be made available on request.
