# Supplementary material for: Deletion of the predicted β-(1,3)-glucan synthase gene bgs1 changes the cell wall composition and impacts growth and fruiting body formation of Schizophyllum commune
Source: Cell Surf. 2026 Oct 16;16:100183. doi: 10.1016/j.tcsw.2026.100183 (PMC13718416; doi:10.1016/j.tcsw.2026.100183)
Supplement: Supplementary file 1 [file mmc1.docx]

**Supplementary Table 1.** List of Agaricomycetes and JGI Mycocosm protein IDs of their type I and type II β-(1,3)-glucan synthases used in the multiple sequence alignment.

| **Species** | **Type I** | **Type II** |
| --- | --- | --- |
| *Agaricus bisporus* | Agabi_varbisH97_2\|226824 | Agabi_varbisH97_2\|183223 |
| *Amanita muscaria* | Amamu1\|71163 | Amamu1\|700249 |
| *Armillaria mellea* | Armmel1\|1184077 | Armmel1\|1129942 |
| *Armillaria ostoyea* | Armosto1\|253484 | Armosto1\|257992 |
| *Boletus edulis* | Boled5\|3638973 | Boled5\|978614 |
| *Climacocystis borealis* | Clibor1\|1262494 | Clibor1\|1180630 |
| *Coprinopsis cinerea* | CopciAmBm1\|446030 | CopciAmBm1\|384955 |
| *Dichomitus squalens* | Dicsqu464_2\|3115347 | Dicsqu464_2\|3022201 |
| *Laccaria bicolor* | Lacbi55996_1\|1136294 | Lacbi55996_1\|1010477 |
| *Lentinus edodes* | Led_CS584_1\|48489 | Led_CS584_1\|363579 |
| *Lepista nuda* | Lepnud1\|1259489 | Lepnud1\|1255676 |
| *Mycena latifolia* | Myclat1\|136785 | Myclat1\|1146555 |
| *Panellus stipticus* | PanstLUM_15\|g3573.t1 | PanstLUM_15\|g1513.t1 |
| *Pisolithus tinctorius* | Pisti2\|127746 | Pisti2\|992924 |
| *Pleurotus ostreatus* | Pleost1\|1204458 | Pleost1\|1158362 |
| *Psilocybe cubensis* | Psicub1_1\|42852 | Psicub1_1\|80643 |
| *Schizophyllum commune* | Schco3\|2628724 | Schco3\|2035630 |
| *Rickenella mellea* | Ricmel1\|811970 | Ricmel1\|740536 |
| *Trametes versicolor* | Traver1\|12337 | Traver1\|13597 |

**Supplementary Table 2.** Expression of *bgs1* and *bgs2* relative to *arf1* in *S. commune* wild type H4-8A, H4-8BΔ*bgs1*, and H4-8BΔ*bgs1::bgs1* grown for 7 days on MM-N agar.

| Strain | *bgs1* | *bgs2* |
| --- | --- | --- |
| H4-8A | 0.73 | 0.82 |
| H4-8BΔ*bgs1* | 0.00 | 0.81 |
| H4-8BΔ*bgs1::bgs1* | 0.77 | 0.83 |

**Supplementary Figure 1.** Fourier-Transform Infrared (FTIR) spectroscopy of the isolated water-soluble polysaccharide fractions of agar cultures of strains H4-8A (blue), H4-8BΔbgs1 (red) and H4-8BΔbgs1::bgs1 (green). Kleijburg et al. (2025) showed that the wild-type FTIR spectrum was fully explained by the presence of schizophyllan. The spectra were normalized to reference peaks at 2993 to 2815 cm− 1 (C). The peaks between 3700 - 2993 cm-1 (A) and 910 – 866 cm− 1 (H) are indicative for OH-stretching bonds and β-glycosidic linkages in the sample. Absorbance at ∼3400 (B), ∼2900 (D), ∼1650 (E), ∼ 1100 (G) and ∼ 900 (I) cm^−1^ and from ∼1500 to ∼ 1200 cm^−1^ (F) are indicative of pure schizophyllan (Hong et al., 2021; Mohammadi et al., 2018).

Hong, T., Yin, J. Y., Nie, S. P., & Xie, M. Y. (2021). Applications of infrared spectroscopy in polysaccharide structural analysis: Progress, challenge and perspective. Food Chem., 12, 100168. https://doi.org/10.1016/j.fochx.2021.100168.

Mohammadi, A., Shojaosadati, S.A., Tehrani, H. J., Mousavim, S. M., Saleh, T., & Khorasani, A. S. (2018). Schizophyllan production by newly isolated fungus Schizophyllum commune IBRC-M 30213: optimization of culture medium using response surface methodology. Ann. Microbiol., 68, 47-62. https://doi.org/10.1007/s13213-017-1316-9.

**Supplementary Figure 2.** Predicted structures of Bgs1 and Bgs2 with the intrinsically disordered region (IDR; in yellow), the glycosyltransferase domain (GT; in green) and the two transmembrane domains (TMD; in purple) that consist of TM helices 1-6 and 7-17, respectively (A). Multiple sequence alignment of the IDRs of 38 Agaricomycete β-(1,3)-glucan synthases shows conserved regions in Type I (orange) and Type II (blue) (B). Type I synthases contain a DPF motif (orange box), whereas type II synthases contain an NPF motif (blue box). Yellow boxes indicate other conserved regions in Type I and Type II.

**c** Overlaid ^1^H-detected spectra of *S. commune* wild type H4-8A,, H4-8BΔ*bgs1,* and H4-8BΔ*bgs1*::*bgs1* strains grown on ^13^C/^15^N-labeled MM-N agar. Dipolar (A, C) and scalar (B, D) hCH spectra of the co-isogenic strains wild type H4-8A (A-D) (blue), H4-8BΔ*bgs1*(A,B) (orange) and H4-8BΔ*bgs1*::*bgs1* (C,D) (purple). Description of the nomenclature used in the NMR assignment can be found in Safeer et al. (2023).
